# Polarized secretion of Leukemia Inhibitory Factor

**DOI:** 10.1186/1471-2121-9-53

**Published:** 2008-09-18

**Authors:** Eric J Hill, Ann B Vernallis

**Affiliations:** 1 School of Life and Health Sciences, Aston University, Birmingham, UK

## Abstract

**Background:**

The direction of cytokine secretion from polarized cells determines the cytokine's cellular targets. Leukemia inhibitory factor (LIF) belongs to the interleukin-6 (IL-6) family of cytokines and signals through LIFR/gp130. Three factors which may regulate the direction of LIF secretion were studied: the site of stimulation, signal peptides, and expression levels. Stimulation with IL-1β is known to promote IL-6 secretion from the stimulated membrane (apical or basolateral) in the human intestinal epithelial cell line Caco-2. Since LIF is related to IL-6, LIF secretion was also tested in Caco-2 following IL-1β stimulation. Signal peptides may influence the trafficking of LIF. Two isoforms of murine LIF, LIF-M and LIF-D, encode different signal peptides which have been associated with different locations of the mature protein in fibroblasts. To determine the effect of the signal peptides on LIF secretion, secretion levels were compared in Madin-Darby canine kidney (MDCK) clones which expressed murine LIF-M or LIF-D or human LIF under the control of an inducible promoter. Low and high levels of LIF expression were also compared since saturation of the apical or basolateral route would reveal specific transporters for LIF.

**Results:**

When Caco-2 was grown on permeable supports, LIF was secreted constitutively with around 40% secreted into the apical chamber. Stimulation with IL-1β increased LIF production. After treating the apical surface with IL-1β, the percentage secreted apically remained similar to the untreated, whereas, when the cells were stimulated at the basolateral surface only 20% was secreted apically. In MDCK cells, an endogenous LIF-like protein was detected entirely in the apical compartment. The two mLIF isoforms showed no difference in their secretion patterns in MDCK. Interestingly, about 70% of murine and human LIF was secreted apically from MDCK over a 400-fold range of expression levels within clones and a 200,000-fold range across clones.

**Conclusion:**

The site of stimulation affected the polarity of LIF secretion, while, signal peptides and expression levels did not. Exogenous LIF is transported in MDCK without readily saturated steps.

## Background

Most cells secrete a variety of cytokines, either continuously or in response to stimulation. The spatial regulation of cytokine secretion is important since it determines which neighbouring cells respond, but little is known about how cytokine secretion in the constitutive pathway is regulated. With their prominent apical and basolateral domains, epithelial cells have been studied extensively for their ability to sort membrane proteins to specific domains. Sorting depends on hierarchical signals and occurs in multiple stages of the secretory pathway [as reviewed in [1-3]]. Lipid raft-associated and independent pathways have been identified for transport from the trans-Golgi to the apical membrane [4]. Less is known about the sorting of secreted proteins. N-glycans and O-glycans as well as proteinaceous patches have been proposed as sorting signals. Lectins may cooperate with lipid rafts in the transport routes [5].

LIF is a secreted, glycosylated cytokine, of the IL-6 family, that exhibits pleiotropic activities in a wide range of tissues and cell types. LIF is produced by epithelial cells during development and during infection and inflammation, including in the intestine [6], the uterus [7] the lung [8], and the kidney [9].

When cultured on permeable supports, Caco-2 cells display the features of differentiated small intestinal enterocytes, forming polarized monolayers with tight junctions. We chose to characterise LIF secretion by Caco-2, since Moon et al. [10] made an intriguing observation about IL-6 secretion in these cells. They reported that IL-6 secretion was higher from the membrane domain (apical or basolateral) that was stimulated with IL-1β than from the non-stimulated domain, suggesting that secretion could be locally facilitated by a pro-inflammatory stimulus. Whether or not LIF is secreted in a similar way is interesting since LIF and IL-6 are related cytokines and may be co-regulated.

We also studied LIF secretion in MDCK, a renal cell line which displays characteristics of distal tubule cells and collecting duct cells [11]. MDCK were selected since the cell line is well suited to exogenous expression. Murine LIF (mLIF) and human LIF (hLIF) are easily distinguished from endogenous canine LIF in ELISAs. Inducible expression systems based on tetracycline-regulated promoters are available for MDCK which allow the effects of different expression levels on secretion to be measured. Few studies have systematically examined the effects of varying expression levels. Marmorstein et al. [12] conducted a study of vascular endothelial growth factor (VEGF_165_) and transforming growth factor β1(TGF-β1) secretion at different expression levels in a retinal pigment epithelial cell line (RPE-J) by varying the amount of adenovirus used to transfect the cells. Their results indicated that these cytokines were secreted in apical pathways that were readily saturated, suggesting specific transporters. For example, VEGF dropped dramatically from 100% apical secretion to 30% apical with increased expression levels. We tested whether this result could be generalized to mLIF and hLIF in MDCK.

Exogenous expression also facilitated study of the contribution of LIF signal peptides to LIF trafficking. Two secreted isoforms of LIF are derived from the murine LIF gene. The isoforms, corresponding to transcripts LIF-D and LIF-M, encode different signal peptides, which in at least some fibroblasts appeared to affect the localization of LIF protein. After cleavage of the signal peptides, the mature murine proteins had the same polypeptide sequence but the LIF-D protein was secreted and freely diffusible; whereas the LIF-M was secreted but remained associated with the extracellular matrix [ECM;  [13]]. LIF-D and LIF-M transcripts are also produced from the hLIF gene [14,15]; however, differences in localization of the products have not been reported. If, as in fibroblasts, LIF-M was associated with the ECM in epithelial cells, LIF-M secretion might be preferentially directed toward the basolateral membrane, where the ECM is present. To test this hypothesis, LIF-M and LIF-D were exogenously expressed in MDCK.

Our results suggest that exogenous LIF is predominantly secreted in an apical direction in MDCK, regardless of signal peptide or expression level, indicating a lack of specific transporters for LIF.

## Results

### Caco-2 secreted LIF

The polarized secretion of a number of cytokines including IL-6 has been described in Caco-2 cells [10]. Secretion of LIF by Caco-2 has not been previously reported. Caco-2 cells were grown as a confluent monolayer in tissue culture flasks for two weeks to allow them to fully differentiate. Conditioned media was then collected over a 48 hour period and assayed for LIF-like activity using a BA/F3-mLIFR-mgp130 assay (hLIF will activate mLIFR/mgp130). The conditioned media displayed a LIF-like activity, which was detectable over background in up to a 1:16 dilution and did not support BA/F3 parental cells (lacking mLIFR/mgp130). A polyclonal antibody raised against eukaryotic human LIF inhibited the proliferative responses by 75% (data not shown). The neutralization by anti-LIF antibodies demonstrates that a large proportion if not all of the activity in the conditioned media is due to hLIF itself and not to other LIFR-dependent ligands.

An hLIF ELISA, which uses antibodies raised against glycosylated hLIF, detected 32 pg/ml hLIF in the Caco-2 conditioned media. This concentration is similar to what was predicted from the BA/F3-mLIFR/mgp130 assays. The ELISA result demonstrates that Caco-2 constitutively secrete small amounts of LIF.

### Caco-2 preferentially secreted LIF from the basolateral membrane

**Figure 1:**
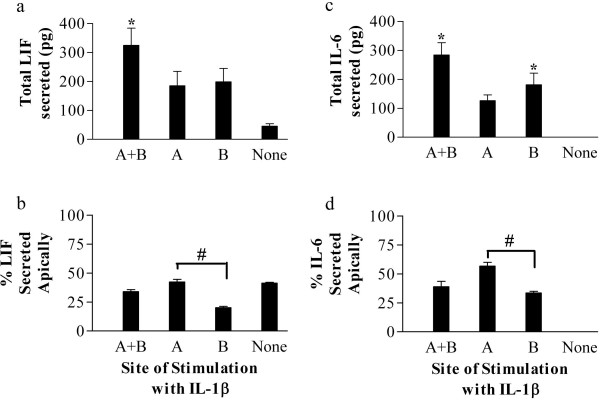
**Caco-2 regulated the direction of secretion of LIF and IL-6 in response to IL-1β**. Caco-2 were grown on permeable supports. a, c) The total amount of LIF and IL-6 secreted into both apical and basal chambers was determined by ELISA 24 hours after addition of IL-1β (1 ng/ml) into either the apical (A), basal (B) or both chambers (A+B). b, d) The percentage of LIF and IL-6 secreted into the apical chamber following treatment. Values represent the mean +/- SEM (n = 3). ND (not detected). *p < 0.05 vs. no stimulation; #p < 0.05 apical vs. basolateral stimulation.

To determine the polarity of LIF secretion, Caco-2 cells were grown on permeable filters. The formation of a sealed monolayer after 14 days was confirmed by measurements of the transepithelial electrical resistance (TEER), which, in unstimulated cells reached approximately, 1,800 Ω.cm^2^. Following conditioning for 24 hours, the media from the apical and basal chambers was analyzed by ELISA. As the volumes recovered from the apical and basolateral chambers differed (0.9 ml vs 1.4 ml), amounts rather than concentrations are reported. Unstimulated Caco-2 cells produced on average 46 pg total LIF protein of which 41% was secreted into the apical chamber (Fig. 1a and 1b). This differential accumulation of LIF is likely to represent genuine differences in secretion since transport of LIF across the monolayer was very limited (data not shown).

### Secretion of LIF and IL-6 by Caco-2 was influenced by the site of stimulation with IL-1β

Apical or basal treatment alone with IL-1β resulted in an increase in the total amount of LIF secreted, but the increase was not statistically significant. Simultaneous treatment of both sides (dual stimulation) resulted in a significant 7-fold increase relative to unstimulated cells (Fig. 1a). Differences between apical and basolateral stimulation were apparent when considering the polarity of secretion. With apical stimulation, 42% of the LIF was secreted apically, whereas with basolateral stimulation only 20% of the LIF was secreted apically, demonstrating that the site of stimulation is important in determining the direction of secretion (Fig 1b). Dual stimulation yielded an intermediate percentage secreted apically. The effect of IL-1β on LIF trafficking is not due to a disruption of the monolayer integrity since stimulation with IL-1β did not have a significant effect on monolayer permeability (data not shown).

The samples collected for LIF secretion were also assayed for IL-6 using an IL-6 ELISA. In contrast to LIF, IL-6 secretion was not detected in untreated cells (Fig. 1c). Apical, basal or dual stimulation with IL-1β all increased IL-6 secretion, but only the basal and dual increases were significant, reaching an average of 284 pg with dual stimulation. When stimulated at the apical membrane, 57% of the IL-6 was secreted into the apical chamber (Fig. 1d). In contrast, basolateral treatment with IL-1β resulted in only 33% secreted apically. With stimulation of both sides of the monolayer, an intermediate percentage was secreted apically. As with LIF, therefore, the polarity of IL-6 secretion was differentially regulated by IL-1β at the apical and basolateral membranes. The two cytokines show a closely related but not identical pattern of secretion.

### MDCK secreted a LIF-like factor from the apical membranes

**Figure 2:**
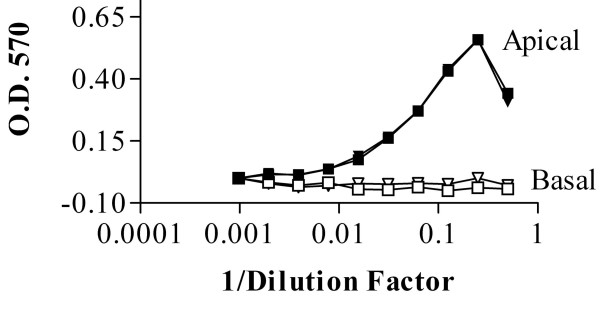
**MDCK apically secreted an endogenous LIF-like factor**. MDCK cells were grown on permeable supports for 24 hours and the conditioned media was collected from the apical and basal chambers. The collected media was then analyzed for LIF-like activity using the BA/F3 mLIR-mgp130 cell assay. Results are expressed as the A_570 _value of cells assayed for proliferation by 3-(4,5-dimethylthiazolyl-2)-2, 5-diphenyl tetrazolium bromide (MTT). Values represent the average activity present in a dilution series of media collected from an individual transwell assayed in triplicate. Two Transwells^® ^are shown with one denoted by a square and one by a triangle. A single representative experiment is shown of n = 3.

The availability of tetracycline-responsive systems (Tet off) for MDCK makes it an attractive cell line for manipulating secretion. Demonstrating that MDCK secreted endogenous LIF in a polarized manner would suggest that the cells actively sort LIF. Studying the secretion of endogenous LIF in MDCK, however, is problematic since antibodies to canine LIF are not available. In lieu of an ELISA, conditioned media from unstimulated MDCK was assayed on BA/F3-mLIFR-mgp130 cells. The media demonstrated a LIF-like activity that was dependent upon LIF-R/gp130 (data not shown). Since the efficiency with which this assay detects the canine-LIF-like activity is unknown, the assay can only measure relative amounts of LIF-like activity. When monolayers of MDCK were grown on permeable filters, and conditioned media was collected after 48 hours, all of the LIF-like activity was detected in the apical compartment (Fig. 2). The apical activity could be diluted up to at least 8-fold and still stay within the linear range of the assay. Taking account of the greater dilution of proteins secreted into the basal compartment, if 20% or more of the LIF-like activity was secreted into the basal compartment, it should have been detected in the bioassay. Stimulating the MDCK cells with human IL-1β did not increase the secretion of the LIF-like activity (data not shown) perhaps because the human cytokine is a weak agonist for the canine receptor [16].

### MDCK mLIF clones secrete LIF primarily from the apical membrane

Since the level of expression has been shown to influence the direction of secretion of growth factors, several stable MDCK Tet-off LIF cell lines were generated for study using the PBI-L expression vector. PBI-L contains a bi-directional promoter, allowing clones to be selected according to their expression of luciferase and/or LIF. To test the polarity of secretion at the minimum and maximum levels of LIF expression, clones were grown on permeable supports and analyzed both in the fully switched on state (no doxycycline; No Dox) or fully switched off (1 μg/ml Dox). Both mLIF and hLIF-expressing clones were generated since differences in their surface-exposed amino acids might result in differences in protein sorting.

As MDCK cells are responsive to stimulation with hLIF [17], the effects of mLIF and hLIF on monolayer integrity were first tested. Eukaryotic mLIF or hLIF (1 ng/ml) was added to both sides of confluent monolayers and the effects on TEER were tested after 24 hours. MDCK cells produced TEERs in the region of 700–850 Ω.cm^2 ^after five days growth on filters. No significant difference in TEER was observed following stimulation with either mLIF or hLIF in comparison to untreated cells. At most small amounts of LIF were transported across the monolayer. After adding LIF to the apical or basal side, less than 5% of the recovered LIF was detected in the opposite chamber.

**Figure 3:**
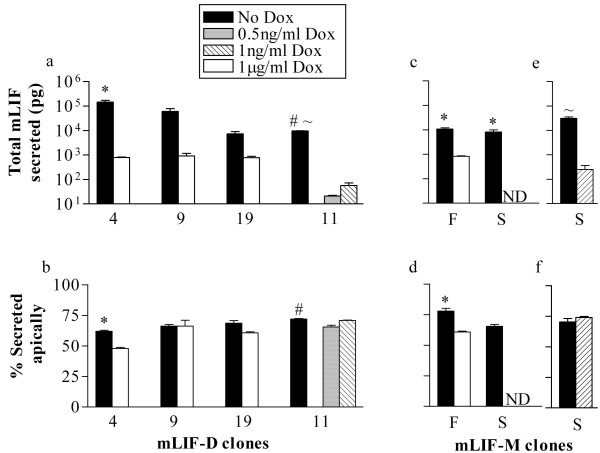
**MDCK Tet-off cells secreted exogenous mLIF-D and mLIF-M predominantly in the apical direction across a range of expression levels**. MDCK cells grown on permeable filters were incubated without doxycycline (No Dox) or varying concentrations of doxycycline (Dox) to regulate the expression level of mLIF-D (a, b) or mLIF-M (c-f). After 24 hours, conditioned media was collected from the apical and basal chambers and analyzed for mLIF by ELISA. a, c, e) Total amount of mLIF secreted. b, d, f) Percentage of mLIF secreted into the apical chamber. Values are expressed as mean +/-SEM (n = 3). ND (not detected at 1 μg/ml Dox). *P < 0.05; vs 1 μg/ml Dox. #P < 0.05; vs. 0.5 ng/ml Dox; ~P < 0.05; vs. 1 ng/ml Dox.

Four **MDCK-Tet-off mLIF-D **clones were analyzed by growing the clones on permeable supports and then assaying the mLIF which accumulated in the apical or basal compartment after 24 hours. Clones 9 and 19 secreted on average 61 and 7 nanograms of mLIF respectively with around 67% of the mLIF secreted apically. They did not differ significantly in their preference for apical secretion at lower expression levels (< 1 ng) observed with 1 μg/ml Dox (63% apical; Fig. 3a and 3b). mLIF-D clone 11 did not produce detectable amounts of mLIF in the presence of 1 μg/ml Dox but with lower amounts of Dox (0.5 ng/ml and 1 ng/ml), mLIF was detected (21 pg and 57 pg respectively). The difference in the percentage secreted apically between No Dox and 0.5 ng/ml Dox is statistically significant, but the difference is small (65% vs. 71%). mLIF-D clone 4, without Dox, secreted 62% apically and with Dox, secreted 48% apically. This 14% drop in the percentage secreted apically was significant but was small compared to the dramatic changes observed for VEGF in RPE-J cells (see discussion). Comparing across expression levels from picograms to nanograms of LIF, there is no compelling evidence for trafficking machinery which can be saturated by mLIF-D.

In order to identify any differences in the pattern of secretion between the LIF-D and LIF-M signal peptides, two **MDCK Tet-off-mLIF-M **clones were also examined over a range of expression levels (Fig 3c–f). Without Dox, mLIF-M clone F produced 11 ng of mLIF (78% in the apical chamber), whereas with Dox this clone produced significantly less mLIF (less than 1 ng) and secreted less of it apically (71%). Without Dox, mLIF-M clone S produced 8 ng of mLIF (66% in the apical chamber), but with 1 μg/ml Dox it did not produce detectable amounts of mLIF. The experiments were repeated with less Dox (Fig. 3e and 3f). In these experiments, clone S secreted without Dox, 31 ng of mLIF (70% in the apical chamber). With 1 ng/ml Dox, clone S secreted significantly less mLIF (253 pg), but secreted a similar percentage apically (74%). Thus of the two clones one showed a difference in the proportion secreted apically at different levels of expression and one did not. Both clones secreted the majority of the mLIF in the apical direction, which is similar to the pattern seen with clones expressing mLIF-D. There is no evidence for a difference between the two signal peptides in directing the polarity of mLIF secretion.

### MDCK hLIF clones also preferentially secrete LIF from the apical membrane

**Figure 4:**
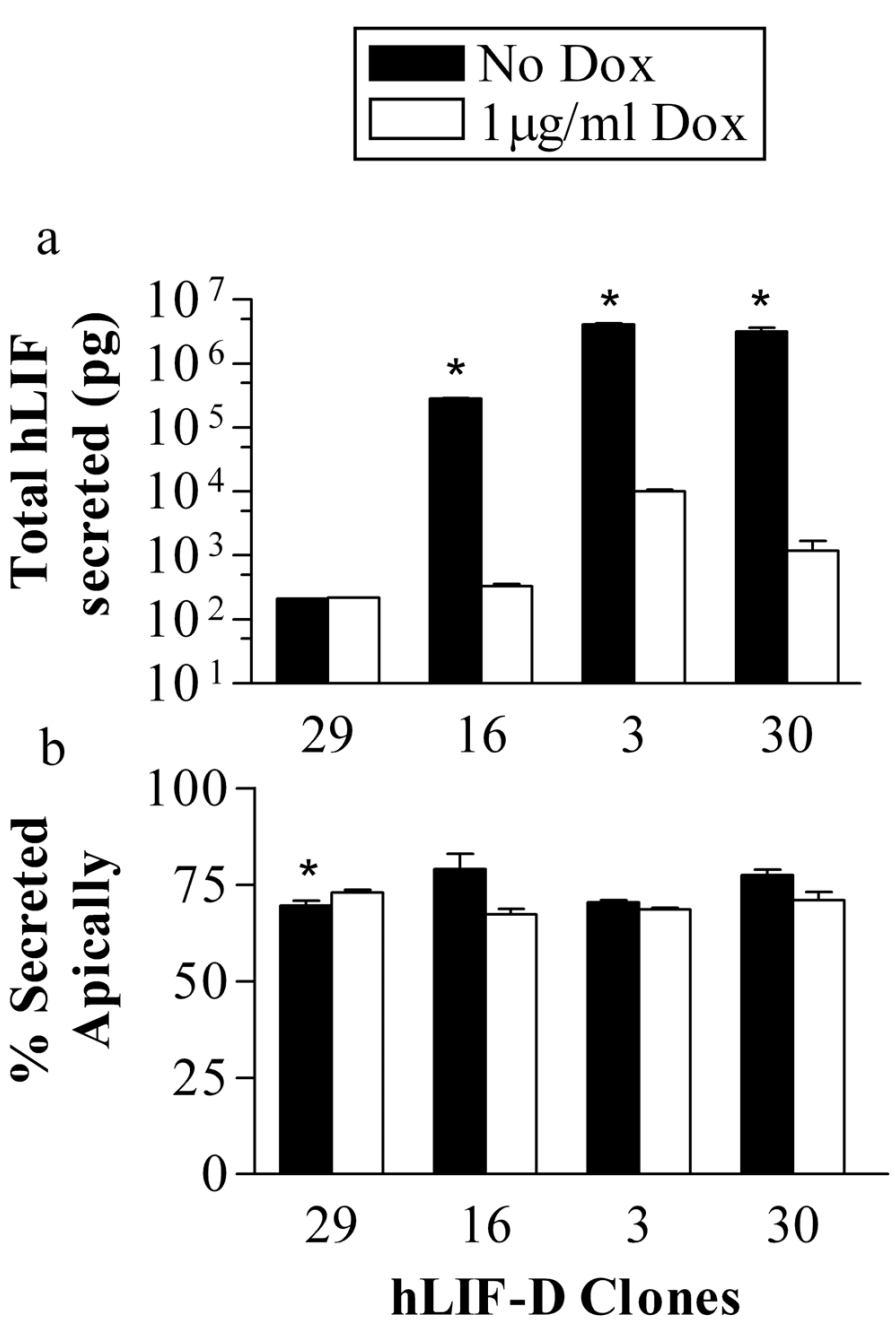
**MDCK Tet-off cells secreted exogenous hLIF-D predominantly in the apical direction across a range of expression levels**. MDCK cells grown on permeable filters were incubated without doxycycline (No Dox) or with 1 μg/ml of doxycycline (Dox) to regulate the expression of hLIF-D. After 24 hours, conditioned media was collected from the apical and basal chambers and assayed for hLIF by ELISA. a) Total amount of hLIF secreted. b) Percentage of hLIF secreted into the apical chamber. Values are expressed as mean +/- SEM (n = 3). *P < 0.05 vs.1 μg/ml Dox.

Four **MDCK Tet-off hLIF-D **clones were also tested for their pattern of secretion (Fig 4). Without Dox, hLIF-D clone 3 produced over 4 μg of hLIF of which 70% of the hLIF was secreted apically. With Dox this clone produced significantly less hLIF (10 ng) but secreted a similar percentage apically (69%). hLIF-D clones 30 and 16 behaved in a similar fashion. Clone 29 produced the least amount of LIF and showed little if any response to Dox. Without Dox, it produced 211 pg of hLIF of which 70% was in the apical chamber, whereas with Dox it produced 220 pg of which 73% was secreted apically. Of the four clones, only clone 29 showed a significant difference in apical secretion but this very small difference (3%) occurred in the absence of a significant change in expression levels of hLIF. Considering the clones together, over a range of concentrations from picograms to micrograms of LIF, the pattern of exogenous hLIF secretion is similar to that of exogenous mLIF in MDCK cells, with about 70% of the LIF secreted apically. There is no evidence for saturation of a sorting step in LIF transport.

## Discussion

The expression of pro-inflammatory cytokines and their receptors by normal (unstimulated) intestinal epithelial cells points towards a role for these cytokines in the normal growth and maintenance of the gut epithelium. The secretion of LIF by unstimulated Caco-2 cells is consistent with the detection of LIF from human colonic epithelial cells [6] and the presence of LIF in mRNA obtained from murine intestinal tissue [18]. Guimbaud et al [19] reported that Caco-2 cells did not make LIF perhaps because they used a less sensitive ELISA. Whilst the human colonic epithelium has been reported to lack LIFR expression, pericryptal fibroblasts express the receptor, suggesting an interaction of LIF produced by colonic epithelial cells with pericryptal fibroblasts [6]. LIF levels are increased in inflammation, as demonstrated by high levels of LIF in patients with ulcerative colitis [19]. In contrast to LIF, we did not observe constitutive secretion of IL-6 from Caco-2. This may reflect different physiological roles for LIF and IL-6 in the normal intestinal epithelium.

It is striking that the site of LIF and IL-6 secretion is influenced by the site of stimulation. Such preferential secretion displays a variety of features. It is observed with some cytokines and acute phase proteins but not all secreted proteins [10,20], with only some inducers [21], and in a variety of polarized cell types [21-24]. The mechanisms which underlie preferential secretion at the site of stimulation have not been defined and merit future study. Enhanced secretion at the site of stimulation could result from a global response such as an increase in the amount of membrane incorporated into the stimulated membrane or a change in membrane composition. If the mechanisms are global, however, there must remain transport routes which are not affected by these changes. For example, Moon et al. demonstrated in Caco-2 that although IL-6 displayed a marked preference for secretion through the membrane stimulated by IL-1β, the secretion of complement component C3 displayed strong basolateral secretion regardless of the membrane stimulated [10].

Conditioned media from MDCK cells demonstrated LIF-like activity in BA/F3 mLIFR-mgp130. LIF expression by MDCK would not be surprising since collecting duct cells are known in rat to express LIFR and LIF. Following ischemia-reperfusion injury LIF is expressed more widely by the epithelial cells of the kidney, where it may participate in regeneration by stimulating the proliferation of epithelial cells [25,26]. LIFR is expressed at both the apical and basolateral membranes of MDCK [17]. The apparent selectivity for apical secretion of a LIF-like activity observed here may reflect paracrine signalling between epithelial cells and is broadly consistent with the apical secretion observed in the exogenous LIF-expressing clones. A sensitive ELISA for canine LIF is required for a more definitive comparison between endogenous and exogenous expression.

We generated cell clones from MDCK Tet-off cells in which LIF expression was regulated by a tetracycline-inducible promoter. The clones demonstrated a wide range of LIF expression levels when the promoters were induced or repressed. Exogenous mLIF was mostly secreted in an apical direction irrespective of whether the signal peptide was from LIF-D or LIF-M. This suggests that the LIF-M signal peptide does not direct the secretion of LIF basolaterally toward the ECM in MDCK. We have not measured the binding capacity of the ECM for LIF in MDCK cells. If the binding capacity is similar to that observed in the osteosarcoma cell line UMR-106 [27], however, it would be easily saturated when LIF is highly expressed in the absence of doxycycline and is unlikely to have affected the measurement of polarity. Cell-type differences may be important in the recognition of sorting signals. When proteins that are polarized in epithelial cells are expressed in fibroblasts, they display specific secretion routes [28,29], however, it is not known if routes used by endogenous proteins in fibroblasts are present in epithelial cells. LIF may also require a specific "sorting escort" as has been observed for components of secretory granules [30].

Interestingly, the level of LIF expression did not influence the proportion of LIF secreted apically and basolaterally. The high levels of LIF produced were comparable to those observed with VEGF when its expression level was manipulated in RPE-J cells [12], but there was no evidence in MDCK of a shift in LIF polarity with increasing expression comparable to the shift observed for VEGF (100% to 30% apical). This failure to observe saturation may reflect a lack of specific sorting receptors for LIF.

The polarity observed with exogenous mLIF and hLIF (70% apical) in MDCK may simply reflect the total volume of vesicles delivered to the apical and basolateral surfaces. This hypothesis is attractive since passive incorporation of LIF into vesicles would account for the lack of saturation. To our knowledge total vesicular traffic has not been measured in MDCK so it is not known what percentage secreted apically would be consistent with passive transport. Comparisons with other four-helical cytokines, however, suggest that intrinsic features of LIF are likely to play a role even in passive transport. Rat growth hormone which is unglycosylated displayed only 35–40% secreted apically when expressed exogenously in MDCK [31,32], indicating differential sorting of LIF and growth hormone. LIF more closely resembles erythropoietin in its secretion pattern. Wild-type erythropoietin displayed predominantly apical secretion when exogenously expressed in MDCK. The percentage apical secretion dropped to about 50% after the removal of one of the N-glycosylation sites [33], suggesting that either the N-glycosylation serves as a sorting signal or it is required for the conformation of a proteinaceous signal. Further experiments could reveal if the glycosylation of LIF was similarly required for apical secretion. One way in which the glycosylation/conformation of LIF could be important is if it mediates a preferential affinity for the lipids that compose apical vesicles or tubules. Constitutive secretory proteins have been shown to be associated with the lumenal face of membranes in the secretory pathway [34]. If LIF can directly interact with lipid domains, it may explain why saturation was not detected for LIF. The high expression levels achieved with exogenous LIF will make experiments with confocal microscopy and detergent solubilization feasible.

The apical secretion of exogenous LIF and an endogenous LIF-like activity in MDCK contrasts with the basolateral preference seen in Caco-2. LIF is not unusual in this regard. Differences in secretion patterns of transmembrane proteins between these two cell lines have been reported and attributed to differences in the recognition of basolateral trafficking signals [35]. We haven't determined whether the preferential secretion of LIF at the site of stimulation is unique to Caco-2 or shared with MDCK. Similarly we haven't determined whether the lack of a role for LIF signal peptides and the absence of saturation are unique to MDCK. For each of these findings, further experiments are required to test whether they extend to both epithelial cell lines.

## Conclusion

The results presented provide evidence for the secretion of LIF by intestinal epithelial cells and a LIF-like activity from kidney epithelial cells. Interesting questions have emerged as to how the site of stimulation is able to influence the pattern of protein secretion and how the secretion of exogenous LIF is directed to the apical and basolateral poles in the apparent absence of specific protein sorters. More mechanistic approaches are now required to pursue these questions.

## Methods

### Cell culture

Caco-2 cells were maintained in DMEM, high glucose, with L-glutamine (Gibco), 20% FCS (Labtech), 1% MEM (Gibco), 100 U/ml penicillin, and 100 μg/ml streptomycin (Gibco). BA/F3 mLIR-mgp130 cells were maintained in RPMI 1640 (Gibco), supplemented with 10% FCS, 50 U/ml penicillin, 50 μg/ml streptomycin, and recombinant human LIF (20 ng/ml). Parental BA/F3 cells were maintained in RPMI 1640 (Gibco), supplemented with 10% FCS, 50 U/ml penicillin, 50 μg/ml streptomycin, and 100 pg/ml IL-3 (R & D systems). MDCK Tet-off cells (BD Biosciences) were maintained in DMEM high glucose containing 50 U/ml penicillin, and 50 μg/ml streptomycin and 1 μg/ml puromycin (Sigma). Polarized secretion experiments were performed with cells grown on 4.7 cm^2^, 0.4 μm pore size polycarbonate membrane Transwell^® ^inserts (Costar). Caco-2 cells were seeded into Transwell^® ^inserts at a density of 4 × 10^5 ^cells/well. Media in each chamber was replaced every two-three days for fourteen days, after which time monolayer integrity was demonstrated by measuring transepithelial resistance (TEER; described below). For stimulation experiments, monolayers were stimulated with 1 ng/ml IL-1β (R & D systems) for 24 hours. MDCK cells were seeded into Transwell^® ^inserts at a density of 10^6 ^cells/well. Media in each chamber was replaced every 2–3 days for five days, after which time monolayer integrity was verified by measuring TEER.

### Epithelial monolayer integrity

The TEER of each monolayer was measured with an epithelial voltmeter using 'chopstick electrodes' (EVOM World Precision Instruments, USA). The mean TEER was calculated from three different positions. The intrinsic resistance of the insert (permeable support alone) was subtracted from the total resistance (cell monolayer and permeable support) to calculate the resistance of the monolayer. The resistance was corrected for surface area of the permeable support (4.7 cm^2^) and the TEER was expressed as Ohms cm^2 ^(Ω.cm^2^). TEER was determined before the start and at the end of each period of collecting conditioned media.

### Proliferation assay

Activity of conditioned media was assessed by a cell proliferation assay. BA/F3 mLIR-mgp130 is a pro-B-cell line that has been stably transfected with cDNA, encoding both components of the mLIF receptor: mLIFR and mgp130. The assay was performed as described previously [36]. Proliferation was measured using 1-(4,5-Dimethylthiazol-2-yl)-3,5-diphenylformazan (MTT; [37]). Neutralization of LIF-like activity was achieved by adding 10 μg/ml of rabbit anti-hLIF (Chemicon; AB1886) to the assay.

### ELISAs

LIF and IL-6 concentrations in cell supernatants were determined by specific ELISA: hLIF, hIL-6 (Bender MedSystems), and mLIF (Quantikine ELISA; R & D Systems). In our experiments, the detection limits for eukaryotic hLIF and hIL-6 were 10 pg/ml. The Quantikine mLIF kit detected bacterial mLIF down to 20 pg/ml. The kit also detected purified glycosylated mLIF that we prepared in eukaryotic cells.

### Construction of LIF expression plasmids

mLIF cDNA encoding the LIF-D signal peptide was isolated from the plasmid pXMT2 mLIF-D [13] using the enzyme EcoR1. The fragment was subcloned into the plasmid PcDNA3.1- (Invitrogen) to create the plasmid PcDNA3-mLIF-D. Murine LIF cDNA encoding the LIF-M signal peptide was isolated from the plasmid pXMT2 mLIF-M [13] using EcoR1 and Xho1. The fragment was subcloned into the plasmid, PcDNA3.1 (-), to create the plasmid PcDNA3.1-mLIF-M. To ensure a comparable 5' end to the mLIF clones, hLIF-D cDNA was generated from a pre-existing PcDNA3.1-hLIF plasmid by PCR. The 5' oligonucleotide was directed to the 5' signal peptide and included a BamH1 site and the 3' oligonucleotide was directed to the 3' end of the LIF coding sequence and included an EcoR1 restriction site. The resulting fragment was subsequently ligated into PcDNA3.1 to create the plasmid PcDNA3.1-hLIF-D. cDNAs encoding the mLIF-D, mLIF-M and hLIF-D signal peptides were isolated from their respective PCDNA3.1 plasmids using the restriction enzymes Nhe1 and HindIII and were subsequently ligated into the plasmid pBI-L (BD Biosciences). All constructs generated by PCR were verified by automated DNA sequencing (Functional Genomics, University of Birmingham).

### Stable expression in MDCK Tet-OFF cells

MDCK Tet-Off cells (BD Biosciences) were transfected with PBI-L-mLIF-D, PBI-L-mLIF-M, and PBI-L hLIF-D using calcium phosphate. Stably transfected cells were selected in media containing 100 ng/ml hygromycin and 1 μg/ml doxycycline and cloned using cloning cylinders (Sigma). Cells expressing exogenous LIF were identified by luciferase activity transcribed from the bi-directional promoter of PBI-L and by LIF ELISA.

### Data Analysis

Results are presented as mean ± standard error of the mean (SEM) of three separate experiments in which duplicate wells were analyzed except where otherwise specified. For comparing total secretion in the presence and absence of doxycycline, unpaired t tests were performed. For comparing proportions, the data was first normalized by taking the arcsine of the square root of the proportion. When more than two results were compared, an analysis of variance (ANOVA) followed by the Tukeys test was performed.

## Authors' contributions

EJH and ABV both contributed to the experimental work and the drafting of the manuscript. Both authors read and approved the final manuscript.
